# Prevalence of Coronavirus Disease 2019 (COVID-19) in Different Clinical Stages before the National COVID-19 Vaccination Programme in Malaysia: A Systematic Review and Meta-Analysis

**DOI:** 10.3390/ijerph19042216

**Published:** 2022-02-16

**Authors:** Jun Wei Ng, Eric Tzyy Jiann Chong, Yee Ann Tan, Heng Gee Lee, Lan Lan Chan, Qin Zhi Lee, Yen Tsen Saw, Yiko Wong, Ahmad Aizudeen Bin Zakaria, Zarina Binti Amin, Ping-Chin Lee

**Affiliations:** 1 Biotechnology Programme, Faculty of Science and Natural Resources, Universiti Malaysia Sabah, Jalan UMS, Kota Kinabalu 88400, Sabah, Malaysia; junweing@outlook.com (J.W.N.); eric_ctj@ums.edu.my (E.T.J.C.); 2 Biotechnology Research Institute, Universiti Malaysia Sabah, Jalan UMS, Kota Kinabalu 88400, Sabah, Malaysia; zamin@ums.edu.my; 3 Queen Elizabeth Hospital, Jalan Penampang, Kota Kinabalu 88200, Sabah, Malaysia; yeeann89@gmail.com (Y.A.T.); henggee@moh.gov.my (H.G.L.); llchan2312@gmail.com (L.L.C.); josiah.loquez@gmail.com (Q.Z.L.); yentsensaw@gmail.com (Y.T.S.); wyiko888@gmail.com (Y.W.); ahaisaza@gmail.com (A.A.B.Z.)

**Keywords:** COVID-19 prevalence, Malaysia, clinical stages, meta-analysis, vaccination program

## Abstract

More than 1.75 million COVID-19 infections and 16 thousand associated deaths have been reported in Malaysia. A meta-analysis on the prevalence of COVID-19 in different clinical stages before the National COVID-19 Vaccination Program in Malaysia is still lacking. To address this, the disease severity of a total of 215 admitted COVID-19 patients was initially recorded in the early phase of this study, and the data were later pooled into a meta-analysis with the aim of providing insight into the prevalence of COVID-19 in 5 different clinical stages during the outset of the COVID-19 pandemic in Malaysia. We have conducted a systematic literature search using PubMed, Web of Science, Scopus, ScienceDirect, and two preprint databases (bioRxiv and medRxiv) for relevant studies with specified inclusion and exclusion criteria. The quality assessment for the included studies was performed using the Newcastle–Ottawa Scale. The heterogeneity was examined with an I^2^ index and a Q-test. Funnel plots and Egger’s tests were performed to determine publication bias in this meta-analysis. Overall, 5 studies with 6375 patients were included, and the pooled prevalence rates in this meta-analysis were calculated using a random-effect model. The highest prevalence of COVID-19 in Malaysia was observed in Stage 2 cases (32.0%), followed by Stage 1 (27.8%), Stage 3 (17.1%), Stage 4 (7.6%), and Stage 5 (3.4%). About two-thirds of the number of cases have at least one morbidity, with the highest percentage of hypertension (66.7%), obesity (55.5%), or diabetes mellitus (33.3%) in Stage 5 patients. In conclusion, this meta-analysis suggested a high prevalence of COVID-19 occurred in Stage 2. The prevalence rate in Stage 5 appeared to be the lowest among COVID-19 patients before implementing the vaccination program in Malaysia. These meta-analysis data are critically useful for designing screening and vaccination programs and improving disease management in the country.

## 1. Introduction

The coronavirus disease 2019 (COVID-19) has emerged as a serious public health concern that has caused more than 218 million confirmed cases and 4.5 million associated deaths globally since December 2019 [1]. The disease originated from the novel severe acute respiratory syndrome coronavirus 2 (SARS-CoV-2). It usually causes mild to moderate respiratory symptoms such as cough, sore throat, and/or fever, but asymptomatic individuals have also been identified, which has added challenges to disease control and prevention. Moreover, some COVID-19 patients could also develop severe illnesses manifested by pneumonia, hypoxemia, multi-organ dysfunction, and acute respiratory distress syndrome (ARDS), which could eventually lead to death. Currently, the results of different studies on the asymptomatic proportion vary significantly from country to country, with at least 1.4% up to 80% [2,3,4,5,6]. On the other hand, among those COVID-19 patients who experience symptoms, about 80% of them have developed mild to moderate symptoms. In comparison, 10–20% of the cases presented with severe symptoms throughout the disease, and about 5–6% have become critically ill with ARDS, multi-organ failure, and/or septic shock [7,8,9].

The virus can be transmitted from one individual through virus-contaminated surfaces and liquid particles ranging from larger respiratory droplets to smaller aerosols [10]. The origin of the SARS-CoV-2 was reported to be possibly zoonotic. The virus is transmitted from an animal, such as a bat, to humans with or without an intermediate host [11]. Several risk factors such as male gender, older age (>60 years old), obesity, and certain health conditions (e.g., hypertension, diabetes, thrombosis, etc.) could affect the severity of COVID-19 [12,13,14,15]. Several types of vaccines, including mRNA-based, viral-based, and inactivated-based, have been developed but are recently reported with a limited ability to reduce the risk and severity of infection owing to the appearance of COVID-19 variants, particularly the Delta variant (B.1.617.2) [16].

As of August 2021, Malaysia has recorded more than 1.75 million infections and 16 thousand deaths since the first reported cases on 25 January 2020. In response to this, the Malaysian government has implemented multiple national lockdowns known as the “Movement Control Order” and “National Recovery Plan” to restrict the spread of COVID-19 in the country. The Ministry of Health (MOH) Malaysia has also developed a COVID-19 Management Guideline (No.5/2020) to manage the COVID-19 cases at designated local hospitals [17]. According to the COVID-19 Management Guideline, the confirmed COVID-19 patients in Malaysia are classified and explicitly managed based on these five categories: Stage 1 (asymptomatic), Stage 2 (symptomatic without pneumonia), Stage 3 (symptomatic with pneumonia), Stage 4 (symptomatic with pneumonia and require supplemental oxygen), and Stage 5 (critically ill with multi-organ involvement).

While the prevalence of COVID-19 has been more frequently reported in the Han Chinese population in China, much less is known about the prevalence rates of this infection in Southeast Asia countries [18,19]. In Malaysia, the National COVID-19 Vaccination Program started in February 2021, while numerous independent studies reported the prevalence of COVID-19 cases even before the vaccination program. However, the findings are inconsistent and fragmented. Moreover, there have been no meta-analyses conducted to systematically evaluate the findings of these studies on the prevalence of COVID-19 cases since the pandemic has started in Malaysia. Therefore, a systematic and comprehensive meta-analysis of all eligible studies was performed to ascertain the prevalence of COVID-19 cases before the vaccination programme in Malaysia. Data from this meta-analysis could add significant value in managing the vaccination programme and treating COVID-19 patients in the country.

## 2. Materials and Methods

### 2.1. Study Subjects and Ethics

Information about age, gender, height, weight, and body mass index (BMI) was initially obtained from a total of 215 admitted COVID-19 patients with written consent in a government-based hospital located in Kota Kinabalu, Sabah, Malaysia, from July 2020 to December 2020. This study was conducted before the National COVID-19 Vaccination Program in the country and was approved by the Medical Research & Ethics Committee, MOH Malaysia (NMRR-20-1785-55933). Certified medical officers assessed the clinical symptoms and the highest severity of the COVID-19 patients, and the patients were grouped into five categories according to the MOH Malaysia guidelines: Stage 1 (N = 25), Stage 2 (N = 39), Stage 3 (N = 74), Stage 4 (N = 68), and Stage 5 (N = 9). The presence of morbidities, namely hypertension, diabetes mellitus, dyslipidaemia, and obesity, was also recorded according to their clinical stages. Data on the severity of these 215 admitted COVID-19 patients were later pooled into a meta-analysis.

### 2.2. Literature Search for Meta-Analysis

Based on the PRISMA guidelines (Table S1), a systemic literature search was carried out up to August 2021 using the online databases of PubMed, Web of Science, Scopus, ScienceDirect, and two preprints databases (bioRxiv and medRxiv) for potential and eligible publications. Advanced search strategies with the following keywords: “prevalence”, “COVID-19”, “coronavirus disease 2019”, “SARS-CoV-2”, “severity”, “Malaysia”, and “Malaysian” were used to discover potential studies. The search strategy is provided in Table S2. In addition, we have also reviewed the references of the retrieved literature to identify any possible relevant studies.

### 2.3. Study Eligibility

All studies containing the clinical characteristics or manifestations of COVID-19 patients were identified. The inclusion criteria for each study had to: (i) be an original study reported in Malaysia; and (ii) clearly state the disease severity stages (1, 2, 3, 4, and 5) that followed the national guidelines established by MOH Malaysia. On the other hand, the exclusion criteria included: (i) study without original data such as reviews, editorials, or communications; and (ii) study using different standards to categorise COVID-19 severity levels.

The information of the identified articles was imported into EndNote (Thompson Reuters, Philadelphia, PA, USA). Duplicates between the databases were removed in the first screening. The articles retrieved were again screened based on the title and abstract to identify eligible studies. The potentially eligible studies were then thoroughly reviewed by two independent researchers. After reviewing the full texts, the eligibility of each study was decided based on the inclusion and exclusion criteria.

### 2.4. Data Extraction

Data including the first author’s name, publication year, and the number of patients based on the COVID-19 severity stages were extracted from all included studies. Two independent investigators performed data extraction. For this meta-analysis, the outcome of interest was the prevalence of COVID-19 patients in Malaysia at each stage of clinical severity.

### 2.5. Quality Assessment and Risk of Bias

The Newcastle–Ottawa Scale was applied to assess the quality of the included articles [20]. The studies were judged based on three broad perspectives: (i) the selection of the study groups, (ii) the comparability of the groups, and (iii) the ascertainment of the outcome of interest. The highest score is 9. A score of 6 or above is considered a high-quality study. A score in the range of 3–5 means medium-quality research, and a score below 3 indicates a poor-quality study. This meta-analysis only includes medium- and high-quality studies.

To objectively evaluate the publication bias of the included studies, the funnel plots and Egger’s tests with a *p*-value < 0.05 as the existence of publication bias were performed. Those tests with a *p*-value > 0.05 were considered not to have publication bias [21].

### 2.6. Statistical Analyses

An independent *t*-test was applied to compare the mean difference in health characteristics of the COVID-19 patients with clinical Stages 2–5 to patients with clinical Stage 1 and was considered statistically significant when the *p*-value < 0.05.

The prevalence of COVID-19 in different stages of severity was calculated for each study using the number of reported cases as a numerator and the total sample size as a denominator. Homogeneity across studies was investigated using an I^2^ index (represented as a percentage) and a Q-test (represented as a *p*-value) that indicated heterogeneity between studies. An I^2^ value > 75% and a Q-test with a *p*-value < 0.1 were regarded as high heterogeneity [22]. If significantly high heterogeneity was observed, a random-effect model was used to combine individual effect sizes to create a pooled COVID-19 prevalence. Otherwise, the heterogeneity was ignored, and a fixed-effect model was utilised. A forest plot was generated to illustrate the prevalence of each study with a 95% confidence interval (95% CI) that contributed to the analysis and the combined prevalence rate. All analyses were performed using the Comprehensive Meta-Analysis version 2 software (Biostat, Inc., Englewood, NJ, USA). A sensitivity analysis was also performed using the same software to assess the stability of the results and investigate each study’s influence by omitting a single study sequentially.

## 3. Results

### 3.1. Characteristics of the Subjects in the Early Phase of the Study

The number of male patients is higher than female patients in Stages 1, 4, and 5. The patients in Stage 1 have the highest mean of age (mean age ± SD = 55.32 ± 14.92) while Stage 3 patients have the lowest mean of age (mean age ± SD = 49.22 ± 13.64). Stage 5 patients had the highest average BMI (mean BMI ± SD = 29.53 ± 5.46) whereas the lowest average of BMI is in Stage 2 patients (mean BMI ± SD = 24.84 ± 5.76). The mean age, height, weight, and BMI of Stage 2 to Stage 5 groups were similar to the Stage 1 group with no significant difference. On the other hand, about two-thirds of the cases had at least one general morbidity. Stage 5 patients with critical illnesses had the highest percentage of hypertension (66.7%), obesity (55.5%), and diabetes mellitus (33.3%). Table 1 shows the descriptive characteristics of the patients.

### 3.2. Study Characteristics of Meta-Analysis

**Figure 1:**
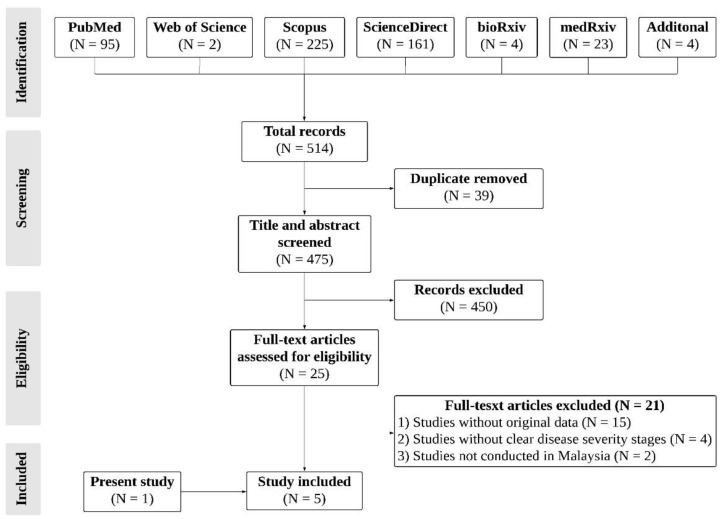
Flow diagram of study selection.

This study has identified a total of 514 records through an initial systematic search of the literature (Figure 1). A total of 475 unique records were screened based on the title and abstract after removing duplications. After extensive screening, 450 records were excluded, and 25 articles were further assessed for eligibility. Among these, 15 articles were excluded due to a lack of original data. Four articles were eliminated due to unclear stages of disease severity. Two articles not reported in Malaysia were removed. Eventually, five studies (four from the databases and the present study) were consistent with the inclusion requirements [23,24,25,26]. Figure 1 illustrates the flow of this study.

Table 2 represents the main characteristics of all five included studies. Three studies were retrospective in design, whereas two were observational studies. The included studies contained 6375 cases showing the number of patients in five different COVID-19 clinical stages admitted in the local hospitals. Moreover, all of the included studies were high quality with a minimum NOS score of 6 (Table S3).

### 3.3. Meta-Analyses Outcomes

**Figure 2:**
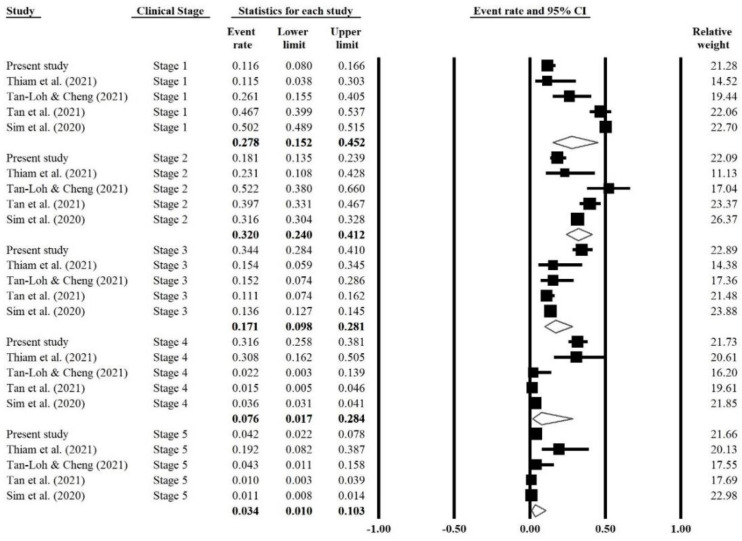
Forest plot of the COVID-19 prevalence grouped according to clinical stages. The black box indicates the prevalence rate of individual studies, and the size of the boxes reflects the relative weight of the study. The error bars indicate the 95% confidence intervals (CI), and the summary effect estimates with their 95% CI are depicted as a diamond. The values in bold are the prevalence rate with its lower and upper limits of each stage.

A significant heterogeneity with an I^2^ > 75% and a *p*-value < 0.1 was observed in all subgroups using a random-effect model (Table 3). In the subgroup analysis based on clinical stages, the forest plot showed that Stage 2 had the highest prevalence rate (32.0%), followed by Stage 1 (27.8%), Stage 3 (17.1%), Stage 4 (7.6%), and Stage 5 (3.4%) (Figure 2).

### 3.4. Publication Bias and Sensitivity Analysis

**Figure 3:**
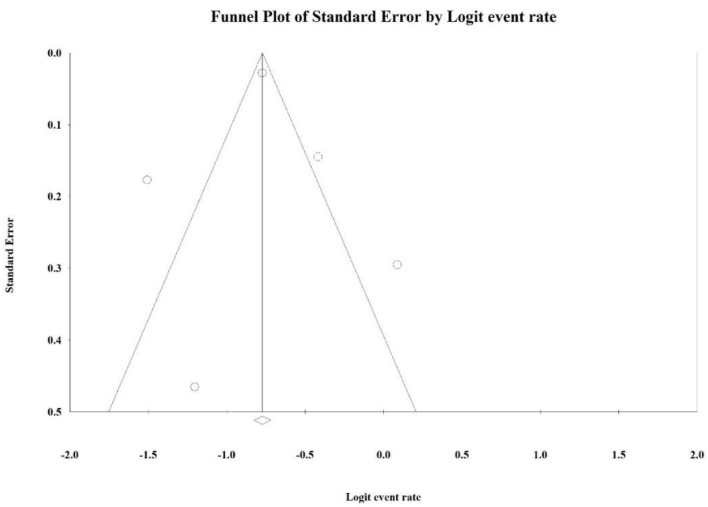
A representative funnel plot of the Stage 2 subgroup of COVID-19 prevalence in this study.

The shape of a representative funnel plot of the Stage 2 subgroup showed a degree of symmetry (Figure 3). Additional statistical evidence from Egger’s tests showed that the overall *p*-value = 0.113, and the *p*-values in all subgroup analyses, were more than 0.05 (Table 3), indicating that the risk of publication bias was low with no systematic differences between all of the studies. There was no publication bias in the meta-analysis.

Sensitivity analysis is used to assess the stability of the pooled results. The prevalence rate and heterogeneity were not significantly altered in all five studies after sequentially omitting each study (Figure S1). In short, the exclusion of each study from the meta-analysis did not substantially influence the results.

## 4. Discussion

Feasible and practical information on the national clinical severity of COVID-19 is greatly needed for establishing better disease management and treatment in Malaysia. The contact- and symptom-based screening might fail to identify all potential SARS-CoV-2 infections, since most cases of COVID-19 were reported to be asymptomatic. The focus of this meta-analysis was to evaluate the prevalence of different clinical stages of COVID-19 before the implementation of the National COVID-19 Vaccination Program in Malaysia. This study is the first meta-analysis to report the prevalence of COVID-19 in Malaysia based on five clinical stages under the guidelines of MOH Malaysia. There is an urgent need to report the prevalence of COVID-19 cases in different stages to design a vaccination programme and effectively manage the country’s national healthcare system to reduce COVID-19 disease-related infection and mortality rates.

Obesity, hypertension, and diabetes mellitus are among Malaysia’s most prevalent health problems [27,28]. Intriguingly, all of these morbidities also serve as the risk factors for severe COVID-19. In this study, obesity, hypertension, and diabetes mellitus were the most common morbidities among COVID-19 patients with critical symptoms, implying that they might play a vital role in developing severe COVID-19. Obese individuals have a higher risk of getting more severe COVID-19, besides having a higher risk for cardiovascular disease, diabetes, and cancer [29,30,31]. In Europe, a high prevalence of obese COVID-19 patients admitted into ICU required invasive mechanical ventilation [32]. The risk for COVID-19 hospitalisation would increase by 14% for every increase of 1 kg/m^2^ in BMI [33], indicating that obese individuals would have higher chances of severe COVID-19. Although the risk of hypertension would increase with age, a retrospective study in Wuhan, China, had shown that hypertension was significantly associated with the severity of COVID-19 even after adjustment of age and gender [34]. The study reported that the odds ratio of hypertension in COVID-19 patients with severe illness was 2.4- to 2.9-fold higher. Severe COVID-19 was observed in diabetes mellitus patients. Diabetic patients were more likely to develop severe COVID-19 conditions due to lower lymphocyte counts and increased serum amyloid A levels [35]. All of these morbidities could act as risk factors that undeniably would impact COVID-19 outcomes.

The COVID-19 severity data that was initially recorded in the early phase of this study was also pooled into a meta-analysis together with another four eligible studies to obtain a precise overview on the prevalence of COVID-19 in different clinical stages before the National COVID-19 Vaccination Program in Malaysia. Overall, this meta-analysis showed that the symptomatic Stage 2 patients who experienced mild symptoms and their courses of infection have the highest prevalence rate (32.0%) in Malaysia. Symptomatic Stage 3 cases with moderate symptoms with the presence of pneumonia consist of 17.1%. These two stages contributed to a majority of about 81% of the total prevalence rate of symptomatic cases in Malaysia. Most people infected with the SARS-CoV-2 virus will experience mild to moderate respiratory illness [10]. A study that included more than 44,000 COVID-19 patients from China reported that 81% of the confirmed COVID-19 cases were mild to moderate [9]. Although the COVID-19 disease pattern in paediatrics might differ from adults, a recent systematic review reported that the mild and moderate cases among the paediatric patients were about 80% of all reported cases [36]. Interestingly, a similar prevalence rate of mild to moderate cases (84%) was also reported in paediatric populations [19]. However, since children usually have a milder presentation of disease symptoms with inconsistent clinical markers [37,38], more severe and better disease precautionary and preventive measures must be established to combat this pandemic.

In general, the mild and moderate cases are isolated at either designated isolation facilities or homes and monitored through their vital signs such as blood oxygen level and body temperature from time to time [39]. Patients are advised to use a pulse oximeter and thermometer for self-assessment. These practices are also applied in managing Stage 2 and Stage 3 COVID-19 patients in Malaysia. Compared to other countries such as China, the application of traditional Chinese medicine or Chinese herbal medicine such as Lianhua Qingwen and Jinhua Qinggan has also been widely studied and proven effective in treating and managing mild to moderate cases [40,41]. An artificial intelligence platform called “IDentif.AI” that can identify effective drug combinations that include a range of antivirals and cancer drugs to treat patients with mild to moderate disease was made available [42,43]. However, in Japan and the United Kingdom, an antibody treatment known as Ronapreve (containing casirivimab and imdevimab) was first approved to treat mild to moderate COVID-19 cases. A global phase III trial found that casirivimab and imdevimab could reduce hospitalization or death by 70% in high-risk non-hospitalised patients and retain activity against emerging variants, including the Delta variant [44]. In short, a majority of the global COVID-19 cases, including in Malaysia, are presented with mild to moderate clinical symptoms. Different countries are utilizing various strategies in treating and managing it with a clear aim to prevent further disease progression.

Since the emergence of COVID-19, asymptomatic cases have always been a problem to disease control in almost every country since they may be contagious and are harder to trace [45]. Even worse, these individuals would be unaware of asymptomatic transmission and therefore normally interact with others and spread the disease unintentionally. A recent study that demonstrated about 59% of all transmissions came from asymptomatic transmission [46]. More importantly, the present meta-analysis raised a vital concern that the prevalence rate of Stage 1 (asymptomatic) COVID-19 patients was the second highest (27.8%) or approximately one-third of COVID-19 cases in the Malaysian population. This prevalence rate is somehow comparable to the number reported in a recent meta-analysis showing that at least one-third of SARS-CoV-2 infections are asymptomatic based on nationwide studies [2]. About one-third of the disease was truly asymptomatic, and the asymptomatic cases were more prevalent in young individuals with no underlying medical condition [3]. However, these data could be underestimated as some asymptomatic cases might be missed and overlooked hence not included in the clinical databases. Additionally, the estimated number of asymptomatic COVID-19 infections in an overall population could be literally three to 20 times higher than the number of asymptomatic cases reported [47]. Furthermore, recent studies have shown a great diverse range of asymptomatic cases [2,3,4,5,6,9]. Asymptomatic cases could threaten national health security. Hence, increasing the capacity of massive COVID-19 community screening to identify and contain the asymptomatic cases is necessary. In addition, more isolation and treatment facilities should be prepared to manage asymptomatic as well as mild and moderate cases, as they have a possible risk of progression to severe illness at any time and ultimately lead to death if not handled well. Other practices such as always wearing a mask in public, social distancing, and avoiding mass gatherings could also stop the spread of asymptomatic cases of COVID-19.

The prevalence of severe and critical COVID-19 cases is an important indicator of the burden on a country’s healthcare system and medical resources, especially intensive care unit (ICU) capacity. For instance, a high ICU load for treating severe COVID-19 cases could give rise to a problem of allocating the ICU for patients with other critical medical conditions. The total prevalence rate of Stage 4 and Stage 5 patients among symptomatic cases in Malaysia was approximately 12% and 5%, respectively. The prevalence rate was similar to the reported data of severely (14%) and critically ill (5%) patients in China’s Han Chinese population study [9]. Moreover, a study in Dakar, West Africa, revealed that about 14.7% of the COVID-19 cases have severe symptoms [48]. A lower percentage was observed in the severe (4.8%) and critical (0.6%) cases in the South Korean population involving 7803 COVID-19 patients and in the Thai population with a low prevalence rate of severe cases at 6.4% [49,50].

On the other hand, a Japanese population-based retrospective study found a much higher prevalence of severe cases. This could be due to the high proportion of senior citizens in Japan [51]. The wide range of prevalence rates in severe and critically ill cases of COVID-19 may be explained by a non-standardized definition of severe COVID-19 in different studies and differences in the study populations in terms of genetics [52,53], sociodemographic, and clinical characteristics. The measures in controlling and managing COVID-19 could affect disease progression and the clinical outcome of the patients. Severe and critically ill COVID-19 cases in the country should be managed immediately and carefully. They have been associated with hypoxemic respiratory failure, ARDS, septic shock, cytokine storm, multiple organ dysfunction, thromboembolic disease, exacerbating underlying comorbidities, and eventually leading to death [54]. More facilities and equipment such as ICU and mechanical ventilators should be readily prepared for Stage 4 and Stage 5 patients. Traditional Chinese medicine therapies which have been reported to be effective against severe COVID-19 disease could be recommended as an alternative therapy for some of the COVID-19 patients in the country to reduce their clinical symptoms [40,41]. Regular COVID-19 disease screening among high-risk communities such as old folks’ homes and dialysis centres should be given attention. These high-risk individuals should be prioritised for vaccination to reduce their exposure to severe COVID-19 disease and death.

There are several limitations to this meta-analysis. Firstly, data presented in this meta-analysis were obtained before implementing Malaysia’s National COVID-19 Vaccination Programme. Since the national vaccination programme, data on the prevalence rate of COVID-19 in Malaysia has not yet been available for comparison. Secondly, other subgroup analyses such as gender, age, ethnicity, lifestyle, comorbidities, and genetic inheritance of the patients were not included due to data limitation and inconsistency. Thirdly, most of the included studies did not clearly state whether the disease severity was only reported as the highest severity level during the course of infection of the COVID-19 patients.

## 5. Conclusions

This study reported the first meta-analysis to investigate the prevalence of COVID-19 in five different clinical stages before implementing the national vaccination programme in Malaysia. The findings revealed that Stage 2 cases have the highest prevalence rate (32.0%) while Stage 5 (3.4%) cases have the lowest prevalence rate. In addition, patients with morbidities such as hypertension, diabetes, and obesity might experience more severe symptoms of SARS-CoV-2 infection. Data from this study may provide an estimation of the burden of COVID-19, which is critical to inform pandemic response and management decisions in Malaysia. Medical practices, public health measures, and a vaccination program should also be vigorously adjusted based on the prevalence rate of COVID-19 in different stages to combat this pandemic in Malaysia.

## Figures and Tables

**Table 1:** Mean difference of health characteristics of COVID-19 patients that were initially recorded in the early phase of this study.

Characteristics	Stage 1 (N = 25)	Stage 2 (N = 39)	Stage 3 (N = 74)	Stage 4 (N = 68)	Stage 5 (N = 9)
	Asymptomatic	Mild	Moderate	Severe	Critical
Gender					
Male	14	19	35	46	6
Female	11	20	39	22	3
Age	55.32 ± 14.92	49.36 ± 15.50	49.22 ± 13.64	51.06 ± 13.15	51.89 ± 9.52
Height (cm)	157.54 ± 9.02	161.88 ± 8.69	161.18 ± 9.78	161.59 ± 6.97	162.00 ± 8.06
Weight (kg)	68.54 ± 12.97	65.18 ± 16.44	71.79 ± 15.60	73.93 ± 16.04	77.00 ± 11.84
BMI (kg/m^2^)	27.46 ± 3.75	24.84 ± 5.76	27.48 ± 4.75	28.23 ± 5.53	29.53 ± 5.46
Morbidities *, N (%)					
Hypertension	11 (44.0%)	14 (35%)	38 (51.4%)	34 (50.0%)	6 (66.7%)
Diabetes mellitus	4 (16.0%)	7 (17.9%)	19 (25.7%)	10 (14.7%)	3 (33.3%)
Dyslipidaemia	4 (16.0%)	5 (12.8%)	15 (20.3%)	10 (14.7)	1 (11.1%)
Obesity (BMI ≥ 30.0)	4 (16.0%)	7 (17.9%)	21 (28.4%)	22 (32.4%)	5 (55.5%)

* Only the number of patients presented with morbidities are shown.

**Table 2:** Characteristics of the literature eligible for meta-analysis in this study.

Study (N = 5)	Study Design	NOS Score	Number of Cases, N (%)					
			Overall	Stage 1	Stage 2	Stage 3	Stage 4	Stage 5
Present study (2021)	Observational	-	215	25 (11.6%)	39 (18.1%)	74 (34.4%)	68 (31.6%)	9 (4.2%)
Thiam et al. (2021) [24]	Retrospective	6	26	3 (11.5%)	6 (23.1%)	4 (15.4%)	8 (30.8%)	5 (19.2%)
Tan-Loh & Cheong (2021) [26]	Retrospective	6	46	12 (26.1%)	24 (52.2%)	7 (15.2%)	1 (2.2%)	2 (4.3%)
Tan et al. (2021) [23]	Retrospective	8	199	93 (46.7%)	79 (39.7%)	22 (11.1%)	3 (1.5%)	2 (1.0%)
Sim et al. (2020) [25]	Observational	7	5889	2956 (50.2%)	1859 (31.6%)	801 (13.6%)	210 (3.6%)	63 (1.1%)

**Table 3:** Meta-analysis of prevalence rates of COVID-19 in subgroups of the study.

Subgroup	Prevalence Rate (95% CI)	Number of Studies	Heterogeneity		Model	Egger’s Test *t*; *p*
			I^2^ (%)	Q-Test		
Stage 1	0.278 (0.152–0.452)	5	96.4	<0.001	Random	2.271; 0.108
Stage 2	0.320 (0.240–0.412)	5	87.7	<0.001	Random	0.044; 0.967
Stage 3	0.171 (0.098–0.281)	5	94.1	<0.001	Random	0.676; 0.548
Stage 4	0.076 (0.017–0.284)	5	98.5	<0.001	Random	0.588; 0.598
Stage 5	0.034 (0.010–0.103)	5	92.0	<0.001	Random	1.699; 0.188

## Data Availability

The data of this study are the included tables, figures, and referenced articles.

## Supplementary Materials

The following are available online at https://www.mdpi.com/article/10.3390/ijerph19042216/s1. Figure S1: Sensitivity analysis of this meta-analysis; Table S1: PRISMA 2020 checklist; Table S2: The search strategy; Table S3: Quality assessment using the Newcastle-Ottawa scale.
